# Exstrophy-Epispadias Complex Variants: A Hybrid Case

**DOI:** 10.3390/pediatric13020024

**Published:** 2021-04-07

**Authors:** Alba Ganarin, Michele Corroppolo, Giosuè Mazzero, Clara Revetria, Fabio Beretta, Enrico Ciardini

**Affiliations:** 1 Pediatric Surgery Unit, Department of Women’s and Children’s Health, University Hospital of Padova, 35128 Padova, Italy; 2 Pediatric Surgery Unit, Santa Chiara Hospital, 38122 Trento, Italy; michele.corroppolo@apss.tn.it (M.C.); giosue.mazzero@apss.tn.it (G.M.); clara.revetria@apss.tn.it (C.R.); fabio.beretta@apss.tn.it (F.B.); enrico.ciardini@apss.tn.it (E.C.)

**Keywords:** exstrophy-epispadias complex, duplicate bladder exstrophy, exstrophy variants

## Abstract

The term exstrophy-epispadias complex refers to a group of midline defects ranging from epispadias to cloacal exstrophy. Bladder exstrophy is the most frequent malformation of this spectrum and it can present as a classical or a variant form. We report a case of a hybrid bladder exstrophy variant having some characteristics of both a duplicate bladder exstrophy and a superior vesical fistula.

## 1. Introduction

Bladder exstrophy incidence is about 2.1–4.0 to 100,000, with its variant ten times more infrequent [1]. The main variants are: pseudoexstrophy, duplicate bladder exstrophy, superior vesical fistula/fissure and covered exstrophy. Duplicate bladder exstrophy is defined as the presence of a normal complete bladder and an exstrophic plate on the anterior abdominal wall [2]. Furthermore, hybrid form existence makes their recognition and classification even more challenging. We here describe a case of a patient having characteristics of two variants. Knowing how to recognize the differences between classical bladder exstrophy and its variants can help the clinician make the correct diagnosis and treatment.

## 2. Case Report

**Figure 1:**
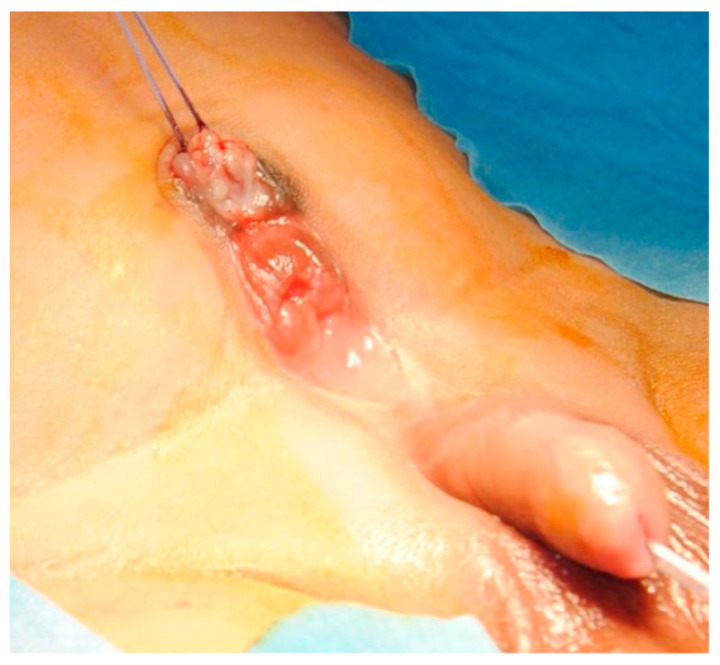
Lower abdomen with exstrophic mucosal plate and normal external genitalia.

A full-term male newborn (birth weight: 3410 g) with a prenatal diagnosis of single umbilical artery presented with an abdominal wall lesion consistent with bladder exstrophy. On physical examination, there was a normally inserted umbilical cord and below it, an abdominal midline defect with a mucosal plate, with no urine leakage (Figure 1). Urine output was normally per-urethral. As no fistulous tract was found after trying cannulation and no methylene blue spillage was noted after retrograde bladder filling, communication between mucosal plate and bladder or ureters was initially excluded. An initial diagnosis of true duplicate bladder exstrophy was therefore made. The patient had normal male external genitalia, normally positioned and conformed anus and no other macroscopic malformations.

**Figure 2:**
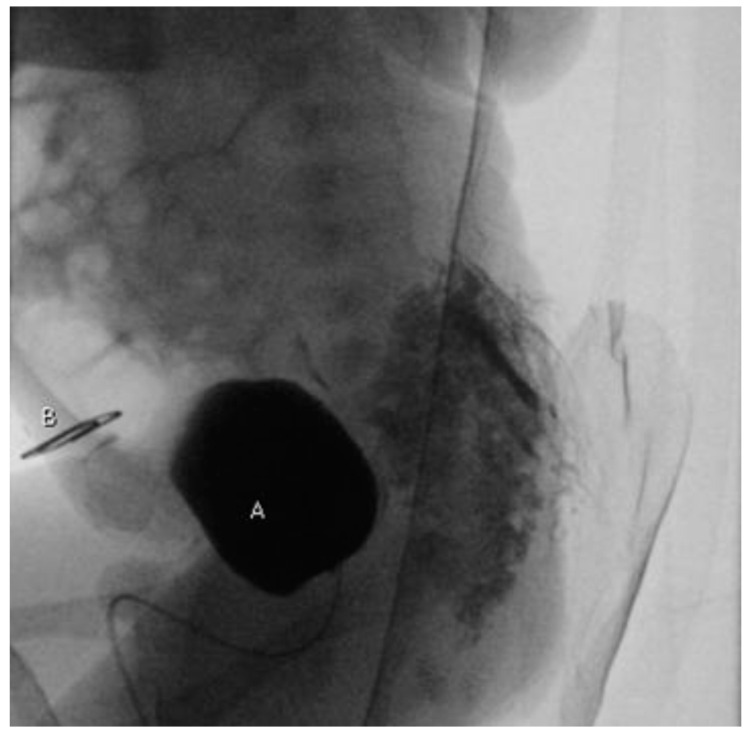
Voiding cystourethrogram (lateral projection): no leakage of contrast medium between bladder (**A**) and exstrophic mucosal plate (**B**) marked with a radiopaque object.

Abdominal ultrasound (US) showed normal kidneys, a left ureter with a 3 mm distal tract dilatation and a normal bladder. A symphysis pubis diastasis was described on plain X-ray. To confirm urinary tract anatomy, also a voiding cystourethrogram was performed (Figure 2): no communication between the normal bladder and the abdominal lesion was documented; a first grade left vesicoureteral reflux was described. During the next days, urine leakage was noted but, even if the everted mucosa was wet, still no fistula was found. In order to clarify the diagnosis (true bladder exstrophy duplication versus superior vesical fistula), we decided to perform a computed tomography urography (CTU): no fistulous tract was documented.

**Figure 3:**
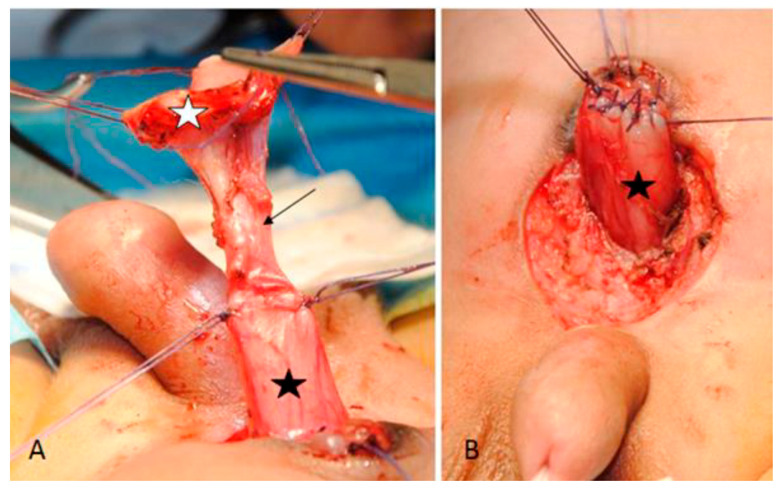
Bladder (black star) exstrophic plate (white star) and their connection (arrow) before (**A**) and after repair (**B**).

At the age of 14 days, the baby was taken to the operating room. An initial cystoscopy showed normal urethra and bladder and a left ectopic ureteral meatus located at the bladder neck. A dot-like solution of continuity was observed at the bladder dome, consistent with a possible urinary fistula. The exstrophic bladder plate was isolated and a tubular connection between this and the normal bladder was found, but no communication between the structures was confirmed. Both the bladder plate and its connection were excised and bladder wall reconstructed (Figure 3). A tension-free abdominoplasty was performed without necessity of an osteotomy; symphysis pubis was approximated with non-absorbable stitches. Post-operative course was uneventful and patient was discharged on the sixth post-operative day. The histopathologic examination was consistent with bladder mucosa and did not describe any fistulous tract.

There were no complications during the outpatient follow-up and urinary tract US, performed after one month from surgical correction, was normal.

## 3. Discussion

The bladder exstrophy-epispadias complex represents an anterior midline defect and includes a wide spectrum of anomalies; its described incidence could be approximated in 1 to 10,000 live births. Bladder exstrophy, one of the possible anomalies, has an incidence of 2.1–4.0 to 100,000 live births [1]. Furthermore, variations of bladder exstrophy are described, representing an even rarer entity, 10 times more infrequent [3]. Pseudoexstrophy is considered the rarest and most benign variant: it is defined as an intact bladder covered with skin and a diastasis of the pubis and rectus muscles. Superior vesical fissure and fistula consist of a sub umbilical bladder communication: depending on defect dimension, the communication could be a fistula (small) or a fissure (larger), being this differentiation arbitrary. In the case of inferior vesical fissure, the bladder dome is intact as there is a communication between lower bladder (above bladder neck) and abdominal wall, with normally formed genitalia. In both pseudoexstrophy and superior vesical fistula/fissure, patients are usually continent because the sphincteric complex is intact. Covered exstrophy is like a classic exstrophy with the same defects, but with a closed and skin-covered bladder [4,5]. Bladder duplication could be side-by-side or anteroposterior, with the latter being considered a true duplicate bladder exstrophy [2]. In this case, there is a closed bladder with normal attached ureters in the pelvis and an exstrophic bladder mucosa on anterior abdominal wall. As there is no communication between the two and the ureters drain in the normal bladder, no urine is present on the everted mucosa. No genital anomaly is present. Surgical treatment consists in everted mucosa excision and primary abdominal closure [6].

Some characteristics of the described patient seemed consistent with the diagnosis of a true bladder exstrophy duplication: exstrophic sub-umbilical mucosal plate, normal enclosed bladder, symphysis pubis diastasis. On the other hand, presence of urine on the everted mucosa, even if no native-bladder communication was documented both radiologically and at surgical exploration, is consistent with a superior vesical fistula. These characteristics make the presented clinical case a probable hybrid form between the two variants of bladder exstrophy. In literature, some other cases similar to ours are reported [7,8].

No alteration of bladder neck or sphincter complex was present in this case, so we expect the patient to be continent. However, he will need urological follow-up not only for incontinence, as for all patients suffering from variants of exstrophy, but also for the first grade left vesicoureteral reflux [2].

## Data Availability

Not applicable.
